# SARS‐CoV‐2 variants circulating in the Fars province, southern Iran, December 2020–March 2021: A cross‐sectional study

**DOI:** 10.1002/hsr2.1373

**Published:** 2023-06-27

**Authors:** Zahra Rezaei, Sadaf Asaei, Shima Sepehrpour, Marzieh Jamalidoust, Mandana Namayandeh, Fatemeh Norouzi, Bahman Pourabbas

**Affiliations:** ^1^ Professor Alborzi Clinical Microbiology Research Center Shiraz University of Medical Sciences Shiraz Iran; ^2^ Department of Microbiology, School of Medicine Fasa University of Medical Sciences Fasa Iran

**Keywords:** Alpha variant, Beta variant, Gamma variant, multiplexed RT‐qPCR, SARS-CoV-2

## 1 INTRODUCTION

Severe acute respiratory syndrome coronavirus‐2 (SARS‐CoV‐2), the causative virus of the COVID‐19 pandemic, claimed millions of lives worldwide and led to deleterious impacts on health services and the global economy. SARS‐CoV‐2 was first reported in December 2019 in Wuhan, China, and has undergone various mutations similar to many RNA viruses. SARS‐CoV‐2 is a single‐stranded positive‐sense RNA virus with a genome of 29,903 nucleotides and 29 proteins that has six major open‐reading frames (ORFs): ORF1a, ORF1b, S (spike), E (envelope), M (membrane), and N (nucleocapsid), and several accessory ORFs.^1^, ^2^, ^3^ Numerous significant variants with further infectious and clinical implications have been discovered globally since the onset of the pandemic.

Previous studies have shown that the virus takes advantage of mutations in the receptor binding domain (RBD) of the spike, the part of the virus that enters human cells, since this makes it easier for the virus to attach to the human angiotensin‐converting enzyme 2 (ACE2) receptor, resulting in increased viral load and infectivity.^4^, ^5^ Variants were classified into a variant of interest (VOI), a variant of concern (VOC), and a variant of under monitoring (VUM).^6^, ^7^ Some variants have higher transmissibility and severity of the disease and, as a result, increase the mortality and morbidity of the disease.^8^ Moreover, created variants can escape the immune system, making the current vaccines ineffective.^9^

Nomenclature system methods were developed by GISAID (https://www.gisaid.org/), Nextstrain, and Pango (Phylogenetic Assignment of Named Global Outbreak) to determine the lineage of SARS‐CoV‐2.^6^ The World Health Organization Virus Evolution Working Group has suggested using the Greek Alphabet, that is, Alpha, Beta, Gamma, and Delta, for each SARS‐CoV‐2 variant to make public communication much more straightforward.^10^

From the onset of the COVID‐19 pandemic until the completion of this study in the middle of March 2021, three variants, including Alpha (B.1.1.7), Beta (B.1.351), and Gamma (P.1), derived from the Wuhan ancestor.^7^, ^10^ Letters and numbers are used to identify mutations, such as D614G, which indicates that amino acid at position 614 of the spike proteins changed from a D (aspartate) to a G (glycine).^8^ Back in March 2020, a significant D614G mutation in the spike glycoprotein of the SARS‐CoV‐2 virus was discovered as a novel variant.^5^, ^11^ This mutation spread to dominate the world, with respective prevalence increased to about 100% by June 2020.^5^, ^11^ In December 2020, Alpha lineage with a vast number of genetic changes, particularly in the spike protein, was detected in the United Kingdom, which continued to expand quickly.^12^ Two VOCs, Beta, and Gamma, also appeared in South Africa and Brazil, respectively, after the dramatic rise of the Alpha variant in the United Kingdom.^13^ These two VOCs then underwent local transmission throughout the world.^13^

All three variants of Alpha, Beta, and Gamma have a mutation at N501Y in which the amino acid asparagine (N) has been replaced by tyrosine (Y) at position 501 in the RBD of the spike protein, making the virus more contagious through facilitating spike protein binding to cellular receptors.^14^, ^15^ In addition, two variants of Beta and Gamma have two additional mutations, K417N/T (substitution of a lysine [K] residue with an asparagine [N] residue or a lysine residue with a threonine) and E484K (substitution of glutamate [E] with lysine [K] at position 484) in the RBD part, which increases the affinity of this part to the ACE2 receptor.^16^

From the reported first case of COVID‐19 in Iran on February 19, 2020 until the commencement of this study, there have been several peaks in the SARS‐CoV‐2 infection, primarily due to the introduction of new variants. This study aimed to determine the SARS‐CoV‐2 variants circulating among individuals by performing multiplexed RT‐qPCR in Fars province, southern Iran.

## 2 MATERIALS AND METHODS

### 2.1 Study design and participants

This cross‐sectional study was conducted on 1895 nasopharyngeal and throat swab samples from individuals who either had symptoms of COVID‐19 or were in contact with people who had COVID‐19 and had been referred to Professor Alborzi Clinical Microbiology Research Center (PACMRC), affiliated with Shiraz University of Medical Sciences for diagnosis between December 21, 2020 and March 17, 2021.

### 2.2 Study area and sample size

Samples of these individuals were taken from six different health centers in Fars province and then transferred to PACMRC. Based on the 11% of COVID‐19 infection rate at the time of the study in PACMRC, a minimal sample size of 151 was estimated (95% confidence interval [CI], 5% margin of error). However, at least 1373 samples were required to achieve such a population, but 1895 samples were tested. Of the 1895 samples, 187 were positive for SARS‐CoV‐2 that 34 were for December 2020, 54 were for January 2021, 52 were for February 2021, and the rest 47 samples for March 2021.

### 2.3 The initial screening and multiplexed RT‐qPCR for variants detection

RNA was extracted from swab samples using the SinaPure™ Virus Extraction Kit (SINACLON). To identify and detect the ORF1ab (RdRp) gene and E gene in novel coronavirus, reverse transcription (RT) and real‐time PCR was performed using the STANDARD M nCoV Real‐Time detection kit by SD BIOSENSOR. Afterward, multiplexed RT‐qPCR was used to screen the SARS‐CoV‐2 Alpha (B.1.1.7), Beta (B.1.351), and Gamma (P.1) variants of concern.^17^ The multiplexed assay detected all three variants by targeting the Δ3675‐3677 spike gene target deletion in the ORF1a gene. Furthermore, by detecting the Δ69/70 HV deletion in the spike gene, the Alpha variant (B.1.1.7) was differentiated from Beta (B.1.351) and Gamma (P.1). To make sure that the target failures are probably due to the presence of the ORF1a and/or spike deletions, CDC N1 (nucleocapsid) primer and probe set was used as a control in this multiplexed to detect both the wild‐type and variant viruses.^18^ The detail of the used primer, probes, and targeted genes are given in Table 1.^17^

**Table 1:** Detail of the used primers and probes.

Target gene	Primer/probe	Sequence
(N1) Nucleocapsid	Fwd primer	GACCCCAAAATCAGCGAAAT
	Rev primer	TCTGGTTACTGCCAGTTGAATCTG
	Probe	FAM‐ACCCCGCATTACGTTTGGTGGACC‐BHQ1
Δ3675‐3677 spike	Fwd primer	TGCCTGCTAGTTGGGTGATG
	Rev primer	TGCTGTCATAAGGATTAGTAACACT
(ORF1a)	Probe	Cy5‐GTTTGTCTGGTTTTAAGCTAAAAGACTGTG‐BHQ2
Δ69‐70 Spike	Fwd primer	TCAACTCAGGACTTGTTCTTACCT
	Rev primer	TGGTAGGACAGGGTTATCAAAC
	Probe	HEX‐TTCCATGCTATACATGTCTCTGGGA‐BHQ1

Multiplexed RT‐qPCR was done using the PCRBIO 1‐Step Go RT‐PCR Kit (PCR Biosystems Ltd) with 200 nM of N1 primers, 100 nM of N1 probe, 400 nM of the ORF1a and spike primers, 200 nM of ORF1a and spike probes, and 5 μL of nucleic acid in a total reaction volume of 20 μL.

Thermocycler conditions included 10 min of reverse transcription at 55°C, 2 min of initial denaturation at 95°C, followed by 40 cycles of 5 s at 95°C and 30 s at 55°C. The distinction between VOCs was made based on ORF1a and/or spike primer‐probe target failure.

### 2.4 Data management and analysis

Data were transferred to SPSS version 18 software (SPSS IBM Corp.) to calculate the frequency of each detected variant. Chi‐square was conducted to compare the frequency of different SARS‐CoV‐2 variants in the samples.

### 2.5 Ethics approval and consent to participate

This study received ethical approval from the Ethics Committee of the Shiraz University of Medical Sciences (SUMS), Shiraz, Iran (No. IR.SUMS.REC.1402.036). Patient's laboratory data were anonymized and deidentified before analysis.

## 3 RESULTS

### 3.1 Prevalence of SARS‐CoV‐2 variants

**Figure 1:**
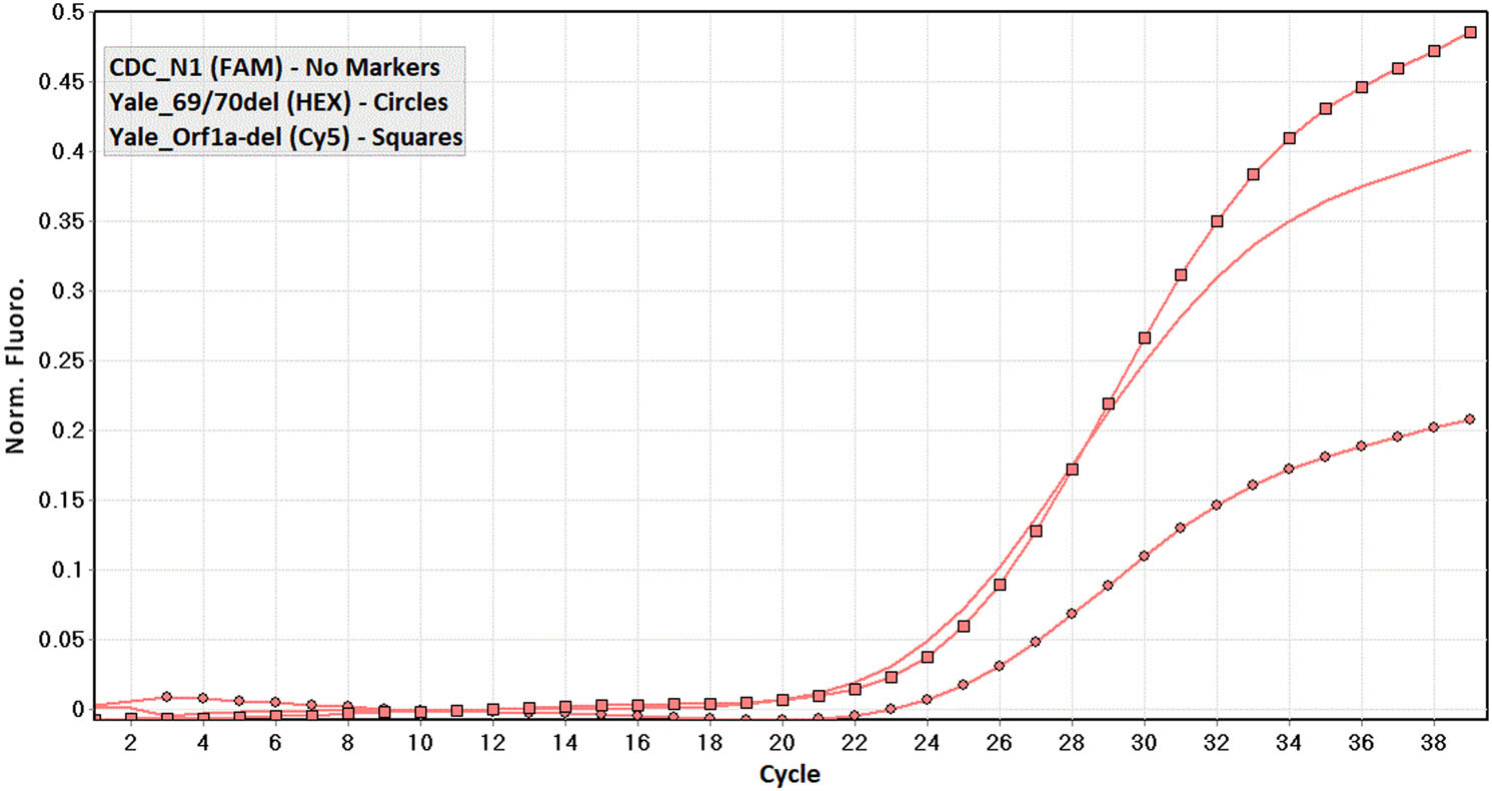
Detection curve related to a sample infected with Wuhan ancestor.

**Figure 2:**
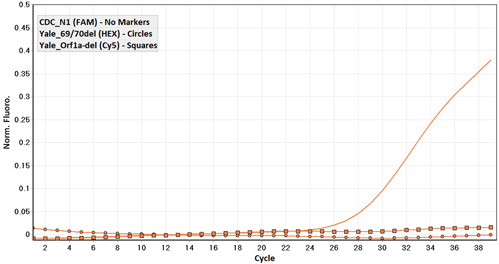
Detection curve related to a sample infected with the Alpha variant.

**Figure 3:**
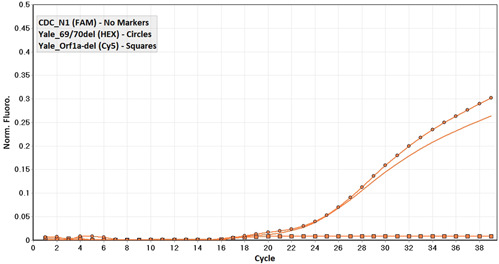
Detection curve related to a sample infected with Beta or Gamma variant.

In the initial screening, out of 1895 samples, 187 were positive for the novel coronavirus. According to the multiplexed RT‐qPCR, out of the 187 positive samples for SARS‐CoV‐2, 74.4% (139/187) belonged to the Wuhan ancestor (Figures 1), 23.5% (44/187) were of Alpha variant (B.1.1.7) (Figure 2), originated from the United Kingdom and 2.1% of the samples (4/187) were Beta (B.1.351) or Gamma (P.1) variants (Figure 3) from South Africa and Brazil, respectively.

All the 34 and 54 samples collected in December 2020 and January 2021 belonged to the Wuhan ancestor, while 67.3% (35) of the 52 samples collected in February 2021 were of the Wuhan ancestor, and the rest, 32.7% (17), belonged to the other variants. Also, of the 47 samples collected in March 2021, 34.1% (16) were from the Wuhan ancestor, and the rest, 65.9% (31), were from other variants.

**Figure 4:**
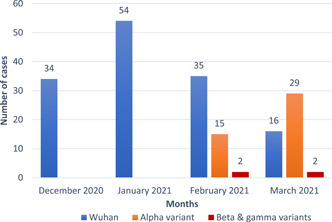
The prevalence of COVID‐19 variants from December 2020 to March 2021 in Fars province.

Of the 32.7% (17/52) variants belonging to February 2021, 88.2% (15/17) were Alpha variants (B.1.1.7), 11.8% (2/17) were the Beta (B.1.351) or Gamma (P.1) variants, while of the 65.9% (31/47) variants belonging to March 2021, 93.5% (29/31) were Alpha variants (B.1.1.7), and 6.5% (2/31) were the Beta (B.1.351) or Gamma (P.1) variants. As it is illustrated in Figure 4, the first entrance of the Alpha, Beta, and Gamma variants was on February 2021.

**Figure 5:**
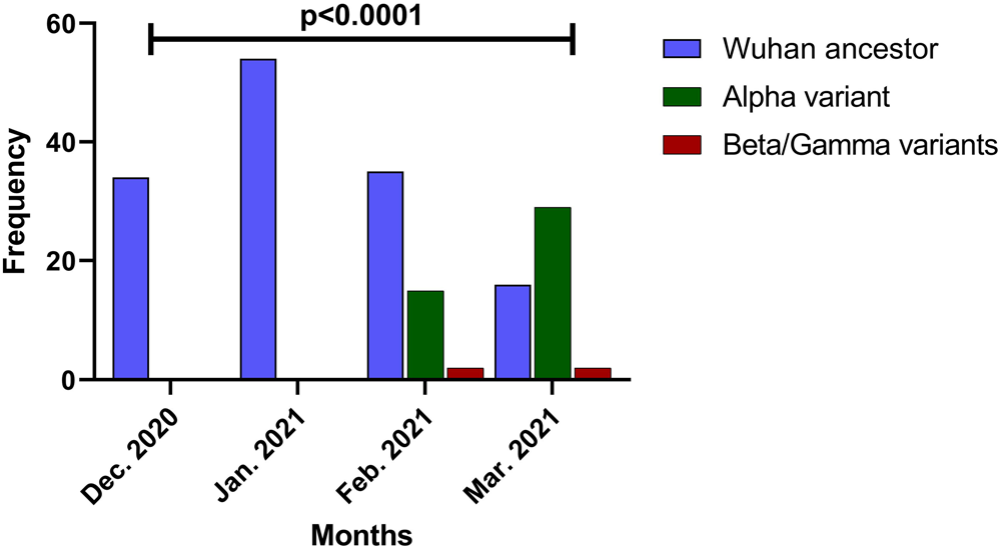
Distribution of severe acute respiratory syndrome coronavirus‐2 variants in Fars province from December 2020 to March 2021.

The *χ* ^2^ test results indicate a significant difference in the frequency of SARS‐CoV‐2 variants across the 4 months (*p* < 0.0001) (Figure 5). Specifically, the Wuhan ancestor variant was more common in December 2020 and January 2021, while the Alpha variant became more prevalent in February and March 2021. The Beta/Gamma variants were also identified in February and March 2021 but in very small frequencies.

## 4 DISCUSSION

There is a possibility of mutation in any virus, including SARS‐CoV‐2, and these mutations can be ineffective or, on the contrary, associated with significant changes in the speed of virus propagation, viral replication, the severity of the disease, their response to the immune system and drugs, and the performance of vaccines. Apart from China, Iran was ranked as one of the first countries with a significant number of COVID‐19 cases. For this reason, the rapid change and evolution of new SARS‐CoV‐2 variants caused great concern about the pandemic. Therefore, it was crucial to monitor the infected individuals in terms of both clinical symptoms and corresponding samples, to detect the emergence of a new variant and, consequently, its impact on the rise or fall in disease cases or severity and ultimately taking essential measures. This study used a multiplexed RT‐qPCR to screen the circulating variants in the community. According to the statistics available on the website: https://ourworldindata.org/coronavirus, the number of individuals with COVID‐19 in Iran in December 21 to 31, 2020, January 2021, February 2021, and March 1 to 17, 2021 were 60,607, 186,570, 206,573, and 245,888, respectively. Accordingly, the deaths in the mentioned months were 1407, 2622, 2035, and 2484, respectively.

Based on the current study findings, the first case of Alpha variants was detected in late February 2021 in Fars province, southern Iran. According to previous studies, the Alpha, Beta, and Gamma variants of SARS‐CoV‐2 are more contagious than the original Wuhan strain.^14^, ^19^, ^20^ Comparing the number of COVID‐19 cases in the current study in February 2021, when the Alpha, Beta, and Gamma variants appeared, with the previous month, January 2021, we found an increase of 20,003 cases, consistent with some reported studies indicating that these variants are more contagious. Similarly, comparing the number of COVID‐19 cases in March 2021, in which about 66% were of the Alpha, Beta, and Gamma variants, with cases in February 2021 revealed an increase of 39,312 cases. Although the study was completed in the middle of March 2021, considering the more transmissibility of these variants, it was predicted that the Alpha, Beta, and Gamma variants would dominate at the end of March 2021, based on the sudden increase of 355,878 cases of COVID‐19 in April (according to the available data on the respective mentioned website). The mortality rate in late December 2020, January 2021, February 2021, and March 1 to 17, 2021 were 2.3%, 1.4%, 1%, and 1%, respectively. But since then, in April, the mortality rate rose again and reached 1.5%. Data from previous studies indicate an increase in the mortality rate of the Alpha, Beta, and Gamma variants compared to the ancestral Wuhan strain.^21^, ^22^, ^23^ COVID‐19 decreased mortality rates in February and March 2021 despite the emergence of Alpha, Beta, and Gamma variants, compared to December 2020 and January 2021, which may be attributed to the national lockdown implemented by the government in late December 2020, vaccination of the healthcare worker and a few elderly people and probably the herd immunity developed in a relative proportion of the individuals exposed to the infection. Since it has been demonstrated that SARS‐CoV‐2 variants may be capable of escaping from neutralizing antibodies and decreasing the antibody efficacy,^24^ identifying the mutants would help quick implementation of control measures to reduce the consequence of the disease.^25^ Although the sensitivity of the RT‐PCR test is not 100% and could be affected by the type of sampling and the items and equipment used, it has remained the mainstay of COVID‐19 diagnosis since the beginning of the outbreak.^26^ In comparison to genome sequencing, the multiplexed RT‐qPCR method is a very suitable, affordable, and straightforward method to identify the variants and, as a result, to determine their origin, the time of their entry to a particular region, and places with high infection rates, thereby taking control measures to prevent the spread of infection.

## 5 CONCLUSION

As revealed, Alpha, Beta, and Gamma variants appeared in late February 2021 in southern Iran. The study highlights the importance of continuous genomic surveillance to monitor the emergence and spread of new variants and their potential impacts on public health. Moreover, this information may assist targeted public health interventions to prevent the spread of COVID‐19 and its variants in the region.

## AUTHOR CONTRIBUTIONS

**Zahra Rezaei**: Conceptualization; formal analysis; investigation; supervision; validation; writing—original draft; writing—review and editing. **Sadaf Asaei**: Data curation; software. **Shima Sepehrpour**: Data curation. **Marzieh Jamalidoust**: Data curation; software. **Mandana Namayandeh**: Data curation. **Fatemeh Norouzi**: Data curation. **Bahman Pourabbas**: Conceptualization; funding acquisition; methodology; project administration; supervision; validation; visualization; writing—review and editing.

## CONFLICT OF INTEREST STATEMENT

The authors declare no conflict of interest.

## TRANSPARENCY STATEMENT

The lead author Bahman Pourabbas affirms that this manuscript is an honest, accurate, and transparent account of the study being reported; that no important aspects of the study have been omitted; and that any discrepancies from the study as planned (and, if relevant, registered) have been explained.

## Data Availability

The datasets related to this article are included within the article. Any additional data will be provided upon request.
